# Candidate genes for anthracnose resistance in Senegalese sorghum: a machine learning-based exploration

**DOI:** 10.1007/s10142-025-01797-6

**Published:** 2025-12-18

**Authors:** Ezekiel Ahn, Insuck Baek, Louis K. Prom, Sunchung Park, Moon S. Kim, Lyndel W. Meinhardt, Clint Magill

**Affiliations:** 1 Sustainable Perennial Crops Laboratory, Agricultural Research Service, Department of Agriculture, Beltsville, MD 20705 USA; 2 Environmental Microbial and Food Safety Laboratory, Agricultural Research Service, Department of Agriculture, Beltsville, MD 20705 USA; 3 Insect Control and Cotton Disease Research, Agricultural Research Service, Department of Agriculture, Southern Plains Agricultural Research Center, College Station, TX 77845 USA; 4 Department of Plant Pathology and Microbiology, Texas A&M University, College Station, TX 77843 USA

**Keywords:** Sorghum, Anthracnose, Machine learning, GWAS, Senegalese germplasm

## Abstract

Anthracnose, caused by the hemibiotrophic fungal pathogen *Colletotrichum sublineola*, is a significant constraint to sorghum production worldwide. Developing resistant cultivars is the most sustainable control strategy, but it requires constant additional sources of resistance genes. Here, we applied machine learning (ML) approaches, specifically Bootstrap Forest and Boosted Tree models, to identify single-nucleotide polymorphisms (SNPs) associated with anthracnose resistance in a panel of Senegalese sorghum accessions using publicly available phenotypic data from seedling and 8-leaf stages. The ML models identified five novel high-importance loci distinct from those found by linear model-based Genome-wide association studies (GWAS), while also reinforcing three candidates detected by both methods. The top candidates found through ML algorithms were leucine-rich repeat (LRR), F-box, aspartic peptidase, and jasmonate O-methyltransferase. Several genes were highlighted by both ML and GWAS, strengthening the evidence for their involvement. This study demonstrates the potential of ML to complement traditional GWAS in identifying candidate genes for complex traits, providing a valuable resource for future functional studies and marker-assisted selection efforts to enhance anthracnose resistance in sorghum. Given the constraints of the available population size, these results are best interpreted as an explanatory framework that highlights potential targets for further investigation and guides future functional validation, rather than as a definitive predictive tool.

## Key message

Machine learning models (Bootstrap Forest, Boosted Tree) applied to Senegalese sorghum phenotypic data identified novel candidate genes for anthracnose resistance (e.g., aspartic peptidase, jasmonate O-methyltransferase) and reinforced candidates like LRR and F-box genes found via single-SNP analysis.

## Introduction

Sorghum [*Sorghum bicolor* (L.) Moench] is the fifth most important cereal crop globally, providing food, feed, and biofuel for millions of people, particularly in semi-arid regions of Africa and Asia (Frederiksen 1986; Yahaya and Shimelis 2022). Sorghum exhibits a good level of tolerance to heat and drought conditions, as well as waterlogged environments, which makes it an ideal crop for cultivation in arid and semi-arid agricultural regions (Yahaya and Shimelis 2022). Like other major cereal crops, sorghum faces ongoing challenges from fungal pathogens that significantly impact both grain yield and quality. (Khaskheli et al. 2025). Among these, anthracnose, caused by the hemibiotrophic fungus *Colletotrichum sublineola*, is one of the most devastating, causing yield losses of up to 50–80% under favorable conditions (high humidity and temperature), and remains a significant yield constraint in semi-arid regions during high-rainfall periods (Aragaw and Terefe 2024; Mekonen et al. 2024). Anthracnose manifests as necrotic spots, stalk rot, and panicle blight, ultimately reducing both grain yield and quality (Mengistu et al. 2018; Stutts and Vermerris 2020; Aslam et al. 2023).

Fungicides are commonly used in developed agricultural systems to control diseases and protect crop yield and quality (Lucas et al. 2015). However, over time, resistance to many effective fungicides has emerged and spread among pathogen populations, undermining disease control (Lucas et al. 2015). Furthermore, the environmental and economic costs associated with fungicide use make them an unsustainable long-term solution. Therefore, the most effective method to achieve long-lasting, stable resistance in cereal crops is to utilize new sources of resistant genes that are naturally found within the plants themselves (Frederiksen 1986; Stuthman et al. 2007; Alsalmo et al. 2025). Stacking multiple resistance genes through molecular breeding, a strategy known as gene pyramiding, is a widely accepted approach to achieving more durable disease resistance in crops (Joshi and Nayak 2010; Mundt 2018; Priyadarshan and Priyadarshan 2019). Combining genes that recognize pathogen effectors or act through different defense pathways makes it possible to significantly reduce the likelihood of pathogen adaptation and resistance breakdown (Joshi and Nayak 2010; Mundt 2018; Priyadarshan and Priyadarshan 2019). This necessitates the identification and incorporation of new resistance genes into top breeding lines as an initial step.

Advances in whole-genome sequencing and statistical methodology have enabled Genome-wide association studies (GWAS), a powerful approach for identifying genetic loci underlying plant complex traits, including disease resistance (Bartoli and Roux 2017; Dubey and Mohanan 2025). GWAS leverages natural genetic variation within a population to identify statistical associations between single-nucleotide polymorphisms (SNPs) and phenotypic traits (Fareed and Afzal 2013; Jin et al. 2015). Previous GWAS efforts have identified several candidate genes for anthracnose resistance in sorghum. For example, analysis of 366 Ethiopian accessions identified *Sobic.009G008800* (encoding a xylem cysteine protease), *Sobic.009G126300* (a threonine-specific protein kinase), and *Sobic.010G012200* (a gluconokinase) as potentially involved in resistance (Mengistu et al. 2021). A separate study using 335 accessions from the Sorghum Association Panel (SAP) implicated genes encoding F-box domain proteins, leucine-rich repeat (LRR), oryzalide A biosynthesis, glucuronosyl transferases, and peroxidases (*Sobic.005G172300*, *Sobic.005G182400*, *Sobic.005G228400*, *Sobic.001G377200*, and *Sobic.001G379400*) (Cuevas et al. 2018). Recent investigation of Senegalese germplasm, focusing on seedling-stage against anthracnose, identified SNPs *Sobic.001G451800* (LRR), S*obic.008G065800* (poly (ADP-ribose) polymerase), and *Sobic.009G162500* (flavonoid 3’-monooxygenase) (Ahn et al. 2022). Moreover, GWAS, based on 8-leaf stage, further expanded the list of candidate genes, including LRR (*Sobic.006G274866*), selenium binding protein (*Sobic.003G401200*), zinc finger (*Sobic.005G194700*), sulfotransferase (*Sobic.005G166700*), protein of unknown function (*Sobic.008G183100*), cytosolic aldehyde dehydrogenase (*Sobic.003G203500*), single strand DNA repair-like protein (*Sobic.002G268900*), and F-box (*Sobic.003G118600*) (Ahn et al. 2021b). While these studies have identified numerous promising candidate genes across diverse sorghum collections and growth stages, they have primarily relied on linear models within the GWAS framework. Such models may not fully capture the complex, non-additive genetic effects, including epistasis, that often underlie quantitative disease resistance. Therefore, a critical need remains a more comprehensive understanding of the genetic architecture of anthracnose resistance, particularly accounting for potential gene-gene interactions.

In recent years, machine learning (ML) algorithms have emerged as a powerful complement to traditional GWAS, offering the potential to capture non-linear relationships and epistatic interactions between genetic variants (Nicholls et al. 2020). ML has been successfully applied to crop species phenotyping and genotype-phenotype association studies. For example, ML combined with hyperspectral imaging has enabled high-throughput phenotyping of cadmium tolerance in jute (*Corchorus* sp.), leading to the identification of candidate genes involved in isoflavonoid biosynthesis and ethylene response pathways (Yang et al. 2024). In sorghum, ML-enabled phenotyping, coupled with GWAS and transcriptome-wide association studies (TWAS), has revealed a complex genetic basis for water-use efficiency (WUE) traits, identifying 394 unique genes enriched for functions related to stomatal development and leaf gas exchange (Ferguson et al. 2021). Furthermore, ML-based GWAS approaches have shown promise in identifying quantitative trait loci (QTL) for yield components in soybean (*Glycine max*), often detecting associations colocalized with previously reported QTLs and supported by functional annotation (Yoosefzadeh-Najafabadi et al. 2022). These studies demonstrate the potential of ML to improve the efficiency and accuracy of identifying genetic determinants of complex traits in crops, including disease resistance.

However, while ML has been used for genomic prediction (Azodi et al. 2019; Dos Santos et al. 2020), its application for marker detection in sorghum remains limited. In this context, we selected Bootstrap Forest and Boosted Tree models because, as ensemble methods, they excel at capturing non-linear genetic interactions and provide interpretable feature importance metrics, offering a distinct advantage over linear models and less interpretable ML algorithms like Neural Networks.

In this study, we hypothesized that ML algorithms, specifically Bootstrap Forest and Boosted Tree models (Breiman 2001; Chen 2014), could identify novel SNPs associated with anthracnose resistance by capturing non-additive genetic effects and complex epistatic interactions that were not identified by a previous GWAS analysis using linear models. We re-analyzed publicly available SNP and phenotypic data from seedling and 8-leaf stage evaluations of a diverse panel of Senegalese sorghum accessions (Ahn et al. 2021b, 2022). We compared the results of ML models to those obtained from a new single-SNP association analysis performed using a series of individual linear regressions. Utilizing the capacity of ML to identify intricate genetic interactions, our goal was to reveal a more comprehensive understanding of the genetic framework related to anthracnose resistance in sorghum, offering important targets for upcoming functional validation and marker-assisted selection. This study will be a solid groundwork for future research in ML-driven SNP mining in future studies, providing extended options of resistance genes against fungal pathogens and eventually contributing to sorghum breeding and sustainable agriculture that impacts millions of people.

## Materials and methods

### Plant material and phenotypic data

The study utilized a diverse panel of Senegalese sorghum landraces. We analyzed publicly available phenotypic and genotypic data representing an overlapping set of germplasm evaluated at two different developmental stages: the seedling stage (*n* = 159) and the 8-leaf stage (*n* = 162). This allowed for a direct comparison of stage-specific resistance mechanisms within the same genetic background. The first study assessed the response of 162 accessions (including susceptible and resistant controls) to a mixture of eight *C. sublineola* isolates (FSP2, FSP5, FSP7, FSP35, FSP36, FSP46, FSP50, and FSP53, sourced from sorghum lines BTx635, RTx2536, and BSBC) at the 8-leaf stage under greenhouse conditions using a spray inoculation method (Ahn et al. 2021b). Using an excised-leaf assay, the second study evaluated 159 accessions at the 1-leaf stage against a single isolate (FSP53) (Ahn et al. 2022). Anthracnose severity was assessed using two distinct 1–5 scales tailored to the specific inoculation method. For the 8-leaf stage greenhouse inoculations, the scale was as follows: 1 = no symptoms or chlorotic flecks; 2 = hypersensitive reaction (HR) without acervuli; 3 = lesions with acervuli on lower leaves; 4 = necrotic lesions with acervuli on lower and middle leaves; 5 = extensive necrosis with abundant acervuli on most leaves, including the flag leaf (Ahn et al. 2021b). For the 1-leaf stage detached leaf assays, the scale was: 1 = no visible fungal infection; 2 = fungal germ tube formation; 3 = fungal growth with incomplete acervuli formation; 4 = 1–5 complete acervuli; 5 = > 5 complete acervuli (Ahn et al. 2022). These two distinct growth stages were specifically selected to investigate ontogenic resistance, allowing for a comparison between early-stage defense mechanisms and those present in more mature plants. This comparative approach is essential for distinguishing stage-specific resistance loci from those conferring durable, broad-stage resistance.

Although the inoculation methods differed (single isolate FSP53 for seedlings vs. a mixture of eight isolates, including FSP53, for the 8-leaf stage), biological consistency in the comparative analysis is supported by the composition of the inoculum. Previous findings indicate that in mixed-isolate infections, the phenotypic response is predominantly determined by the most virulent pathotype present (Ahn et al. 2021a). Given the high virulence of FSP53 (Ahn et al. 2021b), it likely served as the dominant driver of infection in the 8-leaf stage mixture as well. Thus, the comparison effectively screens for resistance against the most aggressive pathotypes present in both stages.

### Genotypic data

The genotypic data consisted of SNPs previously generated by genotyping-by-sequencing (GBS) (Elshire et al. 2011; Upadhyaya et al. 2013; Wang et al. 2013; Hu et al. 2019). The SNPs were aligned to the sorghum reference genome v3.1.1 (Goodstein et al. 2012). To ensure data quality, SNPs with > 15% missing data were first removed. Subsequently, the remaining missing genotype data were imputed using Beagle 4.1 (Browning and Browning 2016). To minimize spurious associations, SNPs with > 15% missing data and a minor allele frequency (MAF) < 0.10 were removed using TASSEL 5 (Bradbury et al. 2007). This filtering resulted in a final set of 99,525 SNPs for subsequent analyses.

### Multivariate analysis

To visualize phenotypic variation and relationships among accessions, t-distributed Stochastic Neighbor Embedding (*t*-SNE) was performed using JMP Pro 17 (SAS Institute Inc., Cary, NC, USA) (Klimberg 2023). Separate *t*-SNE analyses were conducted for the seedling stage, 8-leaf stage, and combined phenotypic data. The following parameters were used for all *t*-SNE analyses: output dimensions = 2, perplexity = 50, maximum iterations = 10,000. Hierarchical clustering was performed using the Ward method based on the filtered SNP data in JMP Pro 17 to assess genetic relationships among accessions with specific options of hybrid goal = 400, hybrid cycles = 30, hybrid initial K = 10, and hybrid RandomPCA Dim = 0.

### Single-SNP association analysis

A single-SNP association analysis was conducted to identify individual SNPs associated with anthracnose resistance at each growth stage. We performed a series of individual linear regressions, with each SNP as a predictor and the anthracnose resistance score as the response variable, using the “Response Screening” platform in JMP Pro 17. This linear approach was employed specifically to establish a baseline for comparison with the non-linear machine learning models. Although the ordinal disease scores (1–5 scale) do not follow a strictly normal distribution, linear regression models were employed as they provide robust approximations for marker effects in GWAS of resistance traits (Prom et al. 2019; Ahn et al. 2021b). To mitigate potential biases from non-normality and control for multiple tests, we applied a False Discovery Rate (FDR) correction (Benjamini and Hochberg 1995). SNPs with an FDR-adjusted *p*-value < 0.01 were significantly associated with anthracnose resistance.

### Machine learning analysis

To identify SNPs associated with anthracnose resistance, we employed a machine learning approach, initially screening several algorithms using the model screening function in JMP Pro 17, including Bootstrap Forest, Boosted Tree, Support Vector Machines (SVM) with a radial basis function (RBF) kernel, and Neural Networks (specifically, Neural Boosted) (Schwenk and Bengio 2000; Breiman 2001; Liu et al. 2011; Chen 2014). These models were chosen for their potential to capture non-linear relationships and interactions between SNPs, which might be missed by traditional linear models used in GWAS. The same filtered SNP and phenotypic data used in the single-SNP association analysis were used for the machine learning analysis. 

Based on iterative preliminary screening to optimize model fit and stability, Bootstrap Forest and Boosted Tree models were selected for their superior explanatory power and interpretability (variable importance scores) compared to other tested algorithms. 

Consequently, we focused on the explanatory power of these two models. Therefore, the final Bootstrap Forest and Boosted Tree models were trained using the entire dataset (100% of accessions). While this approach does not provide an unbiased estimate of predictive accuracy on independent data, it allows us to leverage all available information to identify SNPs with the highest contribution to the model’s fit, as quantified by their importance scores.

For the Bootstrap Forest models, we used the following parameters: number of trees = 100, number of terms sampled per split = 49,278, bootstrap sample rate = 1, minimum splits per tree = 10, maximum splits per tree = 2000, minimum size split = 5. For the Boosted Tree models, we used: number of layers = 71, splits per tree = 4, learning rate = 0.02, minimum size split = 5, row sampling rate = 1.0, column sampling rate = 1.0.

Candidate genes associated with the top-ranked SNPs (based on importance scores) from each model were identified by searching for the nearest/nearby genes within the sorghum reference genome v3.1.1 using the Phytozome 13 database (https://phytozome-next.jgi.doe.gov/*)* (Goodstein et al. 2012).

## Results

### Phenotypic and genotypic clustering of Senegalese sorghum accessions

**Fig. 1:**
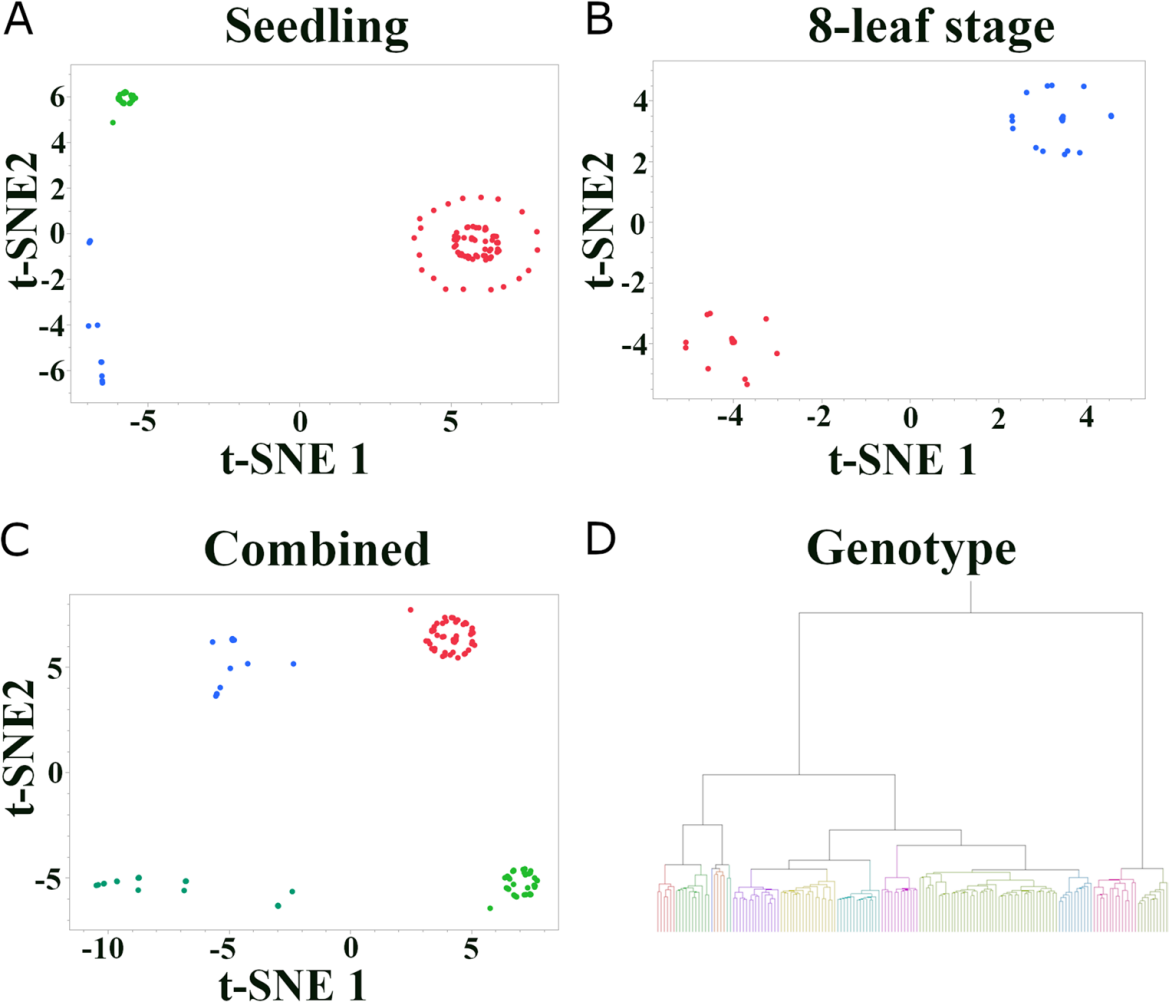
Phenotypic and genotypic differentiation of Senegalese sorghum accessions in response to *C. sublineola*. (A-C) *t*-SNE plots visualizing phenotypic variation in anthracnose resistance: (**A**) Seedling stage resistance to *C. sublineola* isolate FSP53. (**B**) 8-leaf stage resistance to a mixture of eight *C. sublineola* isolates. (**C**) Combined seedling and 8-leaf stage resistance data. Each point represents a single accession, and distances between points reflect phenotypic similarity. (**D**) A hierarchical clustering dendrogram based on 99,525 SNP markers for the 162 unique Senegalese accessions (representing the combined population from both experiments)

*t*-SNE analysis of seedling responses to *C. sublineola* isolate FSP53 revealed three distinct phenotypic groups: one large cluster and two smaller clusters (Fig. 1A). This suggests the presence of different levels of resistance or susceptibility within the seedling population. In contrast, *t*-SNE analysis of the 8-leaf stage responses showed two distinct clusters, positioned on opposite sides of the plot, clearly representing resistant and susceptible phenotypes (Fig. 1B). This suggests a more binary response to anthracnose at the later developmental stage. Combining both seedling and 8-leaf stage phenotypes in the *t*-SNE analysis resulted in four major clusters (Fig. 1C), indicating complex interactions between developmental stage and resistance response.

Hierarchical clustering based on the SNP data grouped the accessions into 13 clusters (Fig. 1D). Notably, two clusters, comprising 24 accessions, were separated from the remaining clusters, suggesting a distinct genetic background for these accessions. This divergence could reflect unique evolutionary histories or adaptation to specific environmental conditions. However, when comparing the anthracnose resistance scores (both seedling and 8-leaf stage) of these 24 accessions to the rest of the population using a two-tailed *t*-test, no statistically significant differences were observed (*p* = 0.41 for seedling and *p* = 0.11 for 8-leaf stage). This indicates that while these 24 accessions are genetically distinct, this overall genetic divergence does not directly translate into a significantly different level of anthracnose resistance as measured in this study.

### Single-SNP association analysis identifies candidate genes for seedling stage resistance

**Fig. 2:**
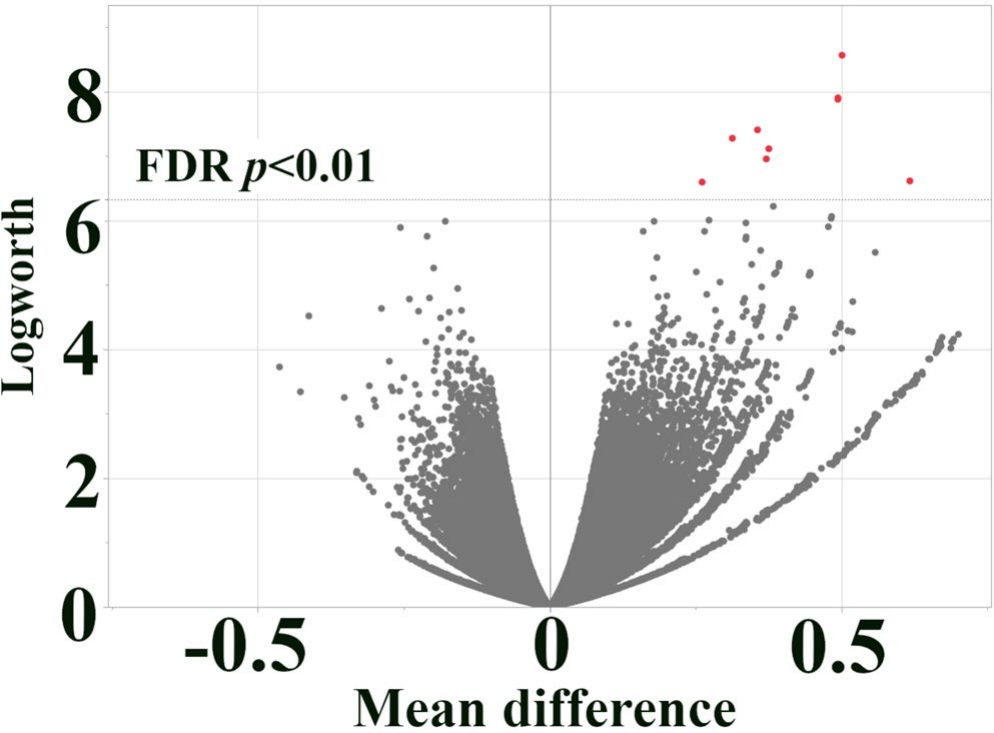
Single-SNP association analysis of seedling anthracnose resistance. Volcano plot of single-SNP association results for seedling resistance to *C. sublineola*. Each point represents a SNP. x-axis: effect size (slope from linear regression). y-axis: -log10(*p*-value). Red points: SNPs with positive effect and FDR-adjusted *p*-value < 0.01. Gray points: SNPs not meeting the FDR significance threshold. Note: Visually distinct peaks may represent clusters of multiple SNPs due to genomic proximity. The exact number of significant SNPs within each locus and their specific chromosomal coordinates are detailed in Tables 1 and 2

**Fig. 3:**
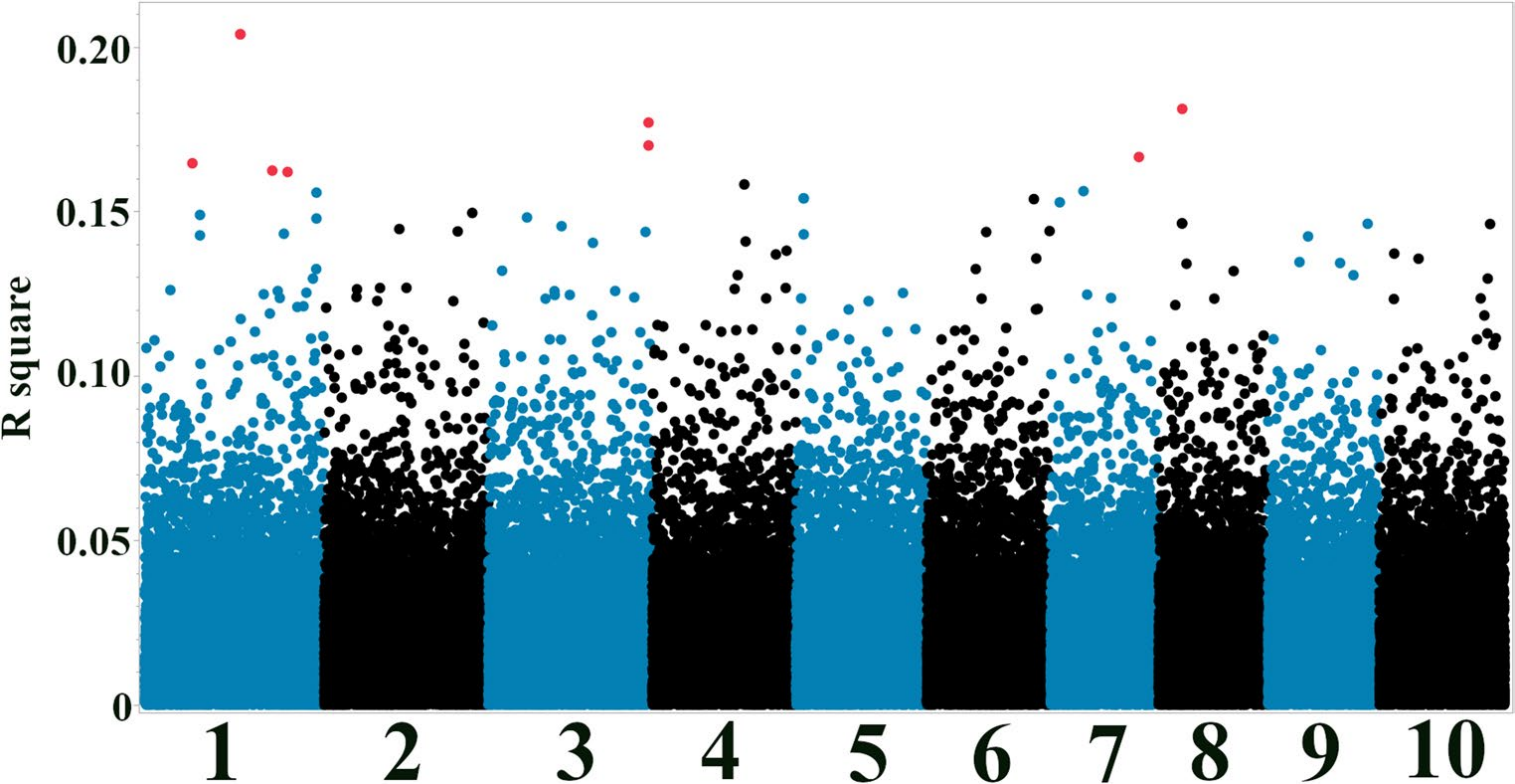
Manhattan plot of seedling anthracnose resistance associations. Manhattan plot of single-SNP association results for seedling resistance to *C. sublineola*. x-axis: SNP position along chromosomes. y-axis: R-squared. Red points: SNPs with FDR-adjusted *p*-value < 0.01. Note: Visually distinct peaks may represent clusters of multiple SNPs due to genomic proximity. The exact number of significant SNPs within each locus and their specific chromosomal coordinates are detailed in Tables 1 and 2

To identify individual SNPs associated with anthracnose resistance, we performed a single-SNP association analysis using linear regression, testing each SNP for a statistically significant association with the phenotypic data from both the seedling and 8-leaf stages. No SNPs were significantly associated with anthracnose resistance at the 8-leaf stage after applying an FDR correction (FDR-adjusted *p*-value < 0.01). However, at the seedling stage, 11 SNPs reached statistical significance (Figs. 2 and 3; Table 1).

**Table 1:** Candidate genes for seedling stage resistance to *C. sublineola* in sorghum identified by single-SNP association analysis. The listed SNPs were significantly associated with seedling stage resistance to *C. sublineola* in 159 Senegalese sorghum accessions (FDR-adjusted *p*-value < 0.01). The table shows the SNP ID, the nearest annotated gene and its putative function, the distance (in base pairs) between the SNP and the gene, and the *p*-value from the single-SNP association analysis. Distance definition: ‘0’ indicates the SNP is located within the gene (intragenic). Values > 0 indicate the distance in base pairs to the nearest annotated gene

SNP ID	Nearby genes and functions	Base pairs away	*p*-value
S1_52723450 S1_52723462 S1_52723482	*Sobic.001G272800* Leucine-rich repeat Disease resistance protein RGA4	13,595	1.89e-8
	*Sobic.001G272700* F-box-like	14,008	
S8_7378286	*Sobic.008G065700* Splicing factor, arginine/serine-rich 2	7,974	1.7e-7
S3_73300553 S3_73300816 S3_73300827	*Sobic.003G430000* Aspartic peptidase	0	2.52e-7
S7_59743196	*Sobic.007G163100* Mediator complex subunit 15 KIX domain-containing protein Mediator of RNA polymerase II	0	6.75e-7
S1_15821946	*Sobic.001G185000* *Leucine-rich repeat* Plant broad-spectrum mildew resistance protein RPW8	2,313	8.06e-7
	*Sobic.001G185100* DUF4005 domain-containing protein	2,100	
S1_64942918	*Sobic.001G359601* Hydrophobic protein RCI2	0	9.93e-7
S1_70039294	*Sobic.001G419600* Heat shock protein 70	0	1.03e-7

These 11 SNPs represent potential candidate genes for anthracnose resistance at the seedling stage. Three of these SNPs (S1_52723450, S1_52723462, and S1_52723482) are located in close proximity to each other on chromosome 1, between *Sobic.001G272800*, which encodes a leucine-rich repeat (LRR) disease resistance protein (RGA4), and *Sobic.001G272700*, which encodes an F-box-like protein (Table 1). LRR proteins are frequently involved in pathogen recognition (Monaghan and Zipfel 2012). F-box proteins are components of the ubiquitin-proteasome system, often playing roles in plant development and environmental responses, including defense signaling (Saxena et al. 2023). Another three significant SNPs (S3_73300553, S3_73300816, and S3_73300827) are located within the coding region of *Sobic.003G430000*, which encodes an aspartic peptidase, a protein family implicated in programmed cell death and defense responses (Chichkova et al. 2014).

Additional significant SNPs were associated with genes encoding diverse functions (Table 1). SNP S8_7378286 is located near *Sobic.008G065700*, encoding a splicing factor (arginine/serine-rich 2). SNP S7_59743196 lies within *Sobic.007G163100*, which encodes a Mediator complex subunit 15 (KIX domain-containing protein), a component of the RNA polymerase II transcriptional machinery. SNP S1_15821946 is located between *Sobic.001G185000*, encoding a leucine-rich repeat protein with homology to the plant broad-spectrum mildew resistance protein RPW8, and *Sobic.001G185100*, encoding a protein with a DUF4005 domain of unknown function. Finally, SNPs S1_64942918 and S1_70039294 are located within the coding regions of *Sobic.001G359601* (encoding a hydrophobic protein, RCI2) and *Sobic.001G419600* (encoding a heat shock protein 70), respectively. These genes represent a diverse range of potential mechanisms contributing to seedling resistance.

### Machine learning identifies candidate SNPs and genes associated with anthracnose resistance

**Fig. 4:**
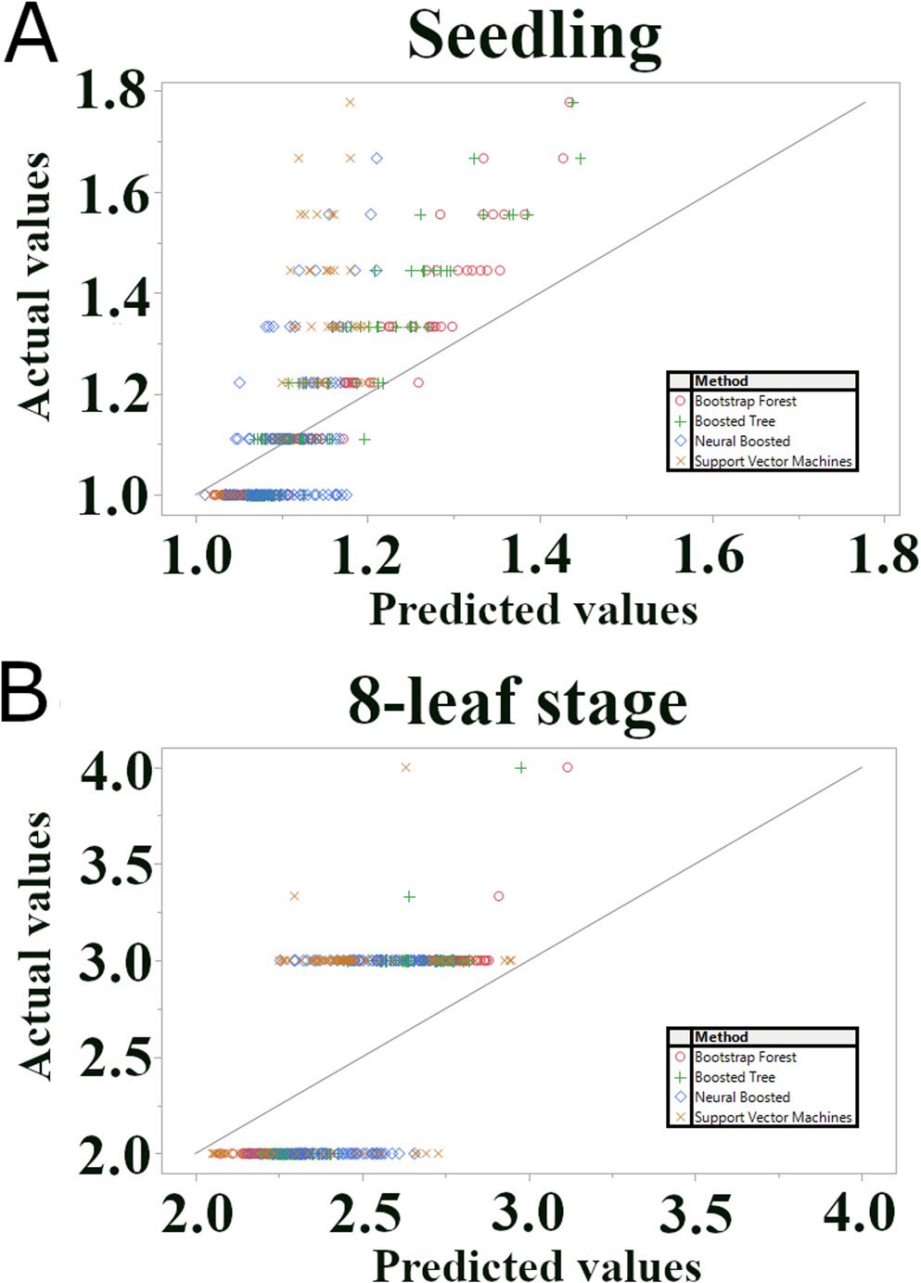
Model fit of machine learning algorithms on the full dataset. Scatter plots of actual vs. predicted anthracnose resistance scores for (**A**) seedling and (**B**) 8-leaf stages. Models (Bootstrap Forest, Boosted Tree, Neural Network, and SVM with RBF kernel) were trained on 100% of the Senegalese sorghum accessions (*n* = 159/162) due to poor validation performance in initial trials. Points represent individual accessions; different symbols represent different models. The diagonal line represents perfect agreement between actual and predicted values

We employed an ML approach to further explore the genetic architecture of anthracnose resistance and identify potential candidate genes beyond those detected by the single-SNP analysis. While other ML algorithms, including Support Vector Machines (SVM) with a Radial Basis Function (RBF) kernel and Neural Networks, were initially considered, preliminary analyses using an 80/20 training/validation split revealed poor predictive performance on the validation set across all models. Specifically, the R-squared values for the validation sets ranged from − 0.32 to 0.31, indicating challenges in generalizing to unseen data, likely due to the high dimensionality of SNP data relative to the limited sample size (*n* = 159/162). Consequently, as justified in the Discussion, we shifted our focus from predictive modeling to explanatory analysis. We trained Bootstrap Forest and Boosted Tree models on the entire dataset (100% of accessions) to identify SNPs with high-importance scores, reflecting their contribution to the model’s fit to the data. The reported R-squared values (0.77 for Bootstrap Forest and 0.60 for Boosted Tree at the 8-leaf stage; 0.78 for Bootstrap Forest and 0.67 for Boosted Tree at the seedling stage) reflect the models’ fit to the entire dataset and should be interpreted as measures of explanatory power, not predictive accuracy (Fig. 4).

**Fig. 5:**
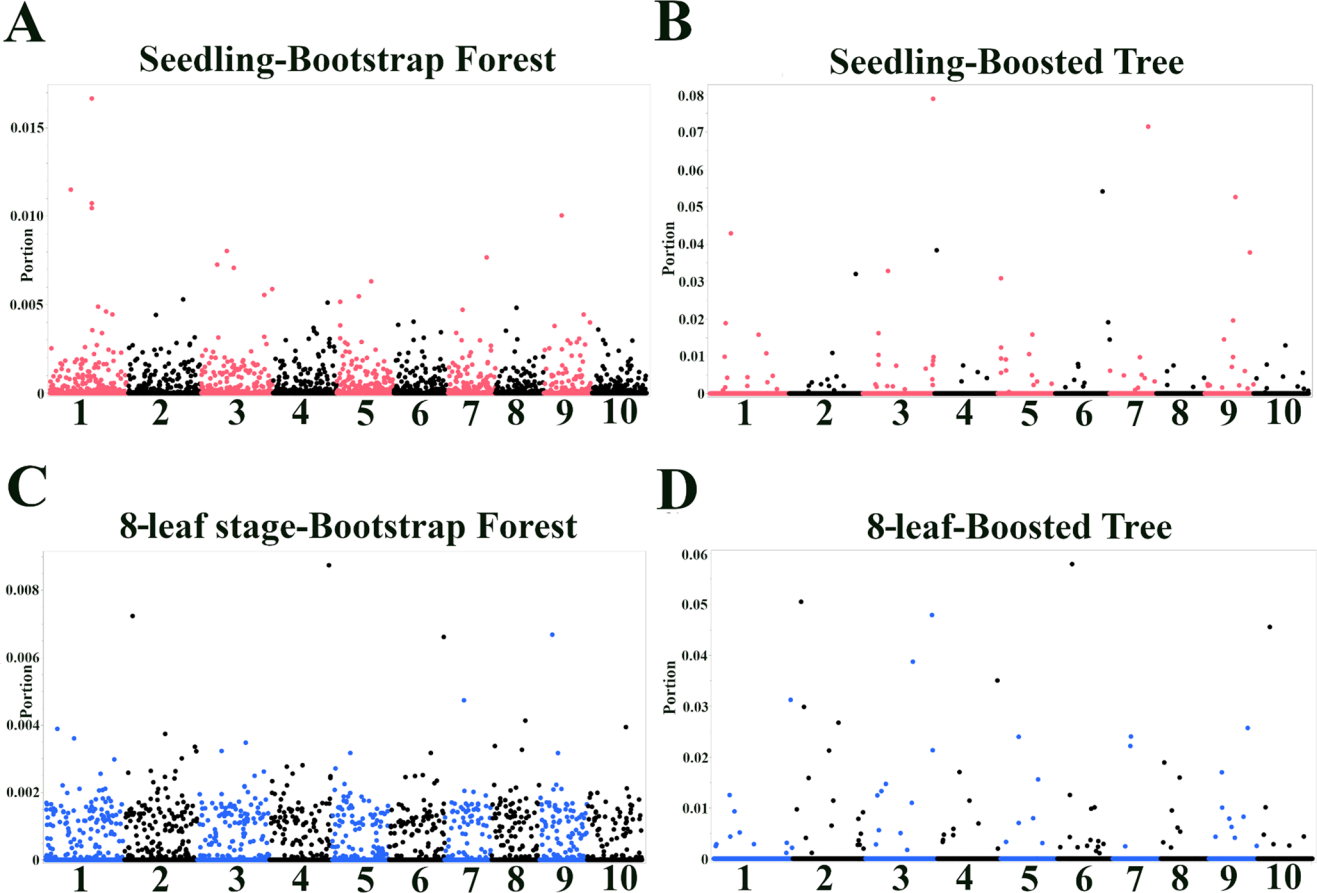
Genome-wide identification of SNPs associated with anthracnose resistance using machine learning. Manhattan plots show results from Bootstrap Forest (**A**, **C**) and Boosted Tree (**B**, **D**) models trained on the complete dataset. Panels (**A**, **B**) depict seedling stage resistance (n = 159), and (**C**, **D**) depict 8-leaf stage resistance (n = 162). Axes: The x-axis represents chromosomal position. The y-axis label ‘Portion’ explicitly denotes the Importance Score (proportion of contribution to model fit). Note: Visually distinct peaks may represent clusters of multiple SNPs in close genomic proximity; the exact number of significant SNPs within each locus and their specific coordinates are detailed in Tables 1 and 2

Analysis with the Bootstrap Forest and Boosted Tree models, which performed the best, revealed SNPs with high importance scores, suggesting their association with anthracnose resistance at the seedling and 8-leaf stages (Fig. 5; Table 2).

**Table 2:** andidate genes for anthracnose resistance in sorghum identified by bootstrap forest and boosted tree models. bootstrap forest and boosted tree models identified top SNPs for seedling and 8-leaf stage resistance to *C. sublineola* in Senegalese sorghum (n = 159/162) and their nearest/nearby annotated genes. Importance scores (proportion of contribution) reflect the relative contribution of each SNP to the model’s fit to the entire dataset (100% training). A threshold of 0.01 was used for bootstrap forest importance scores, and a threshold of 0.05 was used for boosted tree importance scores. For the 8-leaf stage bootstrap forest model, no SNPs exceeded the 0.01 threshold. The top four most important SNPs are listed in this table instead. Distance definition: ‘0’ indicates the SNP is located within the gene (intragenic). Values > 0 indicate the distance in base pairs to the nearest annotated gene

SNP ID	Nearest/nearby gene and function	Base pairs away	Importance score (portion)
Seedling- Bootstrap Forest			
S1_52723450 S1_52723462 S1_52723482	*Sobic.001G272800* Leucine-rich repeat Disease resistance protein RGA4	13,595	0.017
	*Sobic.001G272700*F-box-like	14,008	
S1_15821946	*Sobic.001G185000* Leucine-rich repeat Plant broad-spectrum mildew resistance protein RPW8	2,313	0.012 Threshold = 0.01
	*Sobic.001G185100*DUF4005 domain-containing protein	2,100	
Seedling- Boosted Tree			
S3_73300553	*Sobic.003G430000* Aspartic peptidase	0	0.079
S7_59743196	*Sobic.007G163100* Mediator complex subunit 15 KIX domain-containing protein Mediator of RNA polymerase II	0	0.072
S6_57126382	*Sobic.006G225800* F-box domain	0	0.054
S9_47938177	*Sobic.009G126000* Mannosylglycoprotein endo-beta-mannosidase/Endo-beta-mannosidase	0	0.0526
8-leaf stage- Bootstrap Forest			
S4_66737769	*Sobic.004G335300* Uncharacterized protein	6,896	0.0087
S2_5624818	*Sobic.002G058100* Thioredoxin domain-containing protein	0	0.0072
	*Sobic.002G058200* Jasmonate O-methyltransferase	5,218	
S9_10884727	*Sobic.009G080233* Uncharacterized protein	24,006	0.0067
S6_60609133	*Sobic.006G274866* Leucine Rich Repeat (LRR_1)//Protein tyrosine kinase (Pkinase_Tyr)//Leucine rich repeat N-terminal domain (LRRNT_2)	4,656	0.0066
8-leaf stage- Boosted Tree			
S6_21593433	*Sobic.006G040401* Zinc finger, CCHC-type	162,184	0.058
S2_5624818	*Sobic.002G058100* Thioredoxin domain-containing protein	0	0.051
	*Sobic.002G058200*Jasmonate O-methyltransferase	5,218	

For seedling resistance, the Bootstrap Forest model showed a prominent peak on chromosome 1 (Fig. 5A). The top SNPs within this peak (S1_52723450, S1_52723462, and S1_52723482) were also identified as significant in the single-SNP association analysis, providing converging evidence for their importance. These SNPs are located between *Sobic.001G272800* (encoding an LRR disease resistance protein, RGA4) and *Sobic.001G272700* (encoding an F-box-like protein), genes with well-established roles in plant defense. Another highly ranked SNP by the Bootstrap Forest model, S1_15821946, is located near *Sobic.001G185000*, encoding another LRR protein with homology to the broad-spectrum mildew resistance protein RPW8 (Xiao et al. 2001). In contrast, the Boosted Tree model for seedling resistance did not show a single dominant peak. Still, the top SNPs were located within the coding regions of genes with diverse functions (Fig. 5B; Table 2): *Sobic.003G430000* (aspartic peptidase), *Sobic.007G163100* (Mediator complex subunit 15), *Sobic.006G225800* (F-box domain protein), and *Sobic.009G126000* (mannosylglycoprotein endo-beta-mannosidase).

At the 8-leaf stage, the Bootstrap Forest model identified several SNPs with notable importance scores (Fig. 5C; Table 2). SNP S4_66737769 is located near *Sobic.004G335300*, an uncharacterized protein. SNP S2_5624818 is located within *Sobic.002G058100* (thioredoxin domain-containing protein) and is also close to *Sobic.002G058200* (jasmonate O-methyltransferase), suggesting potential roles in redox regulation and jasmonate signaling, respectively. SNP S6_60609133 is located near *Sobic.006G274866*, which encodes a protein with LRR and was also detected as a top candidate from a previous study. This protein contains protein tyrosine kinase, and LRR N-terminal domains, suggesting a potential role in signal transduction. The association of these SNPs with nearby genes is supported by the LD decay patterns previously established for this specific Senegalese population, which indicates rapid LD decay (Ahn et al. 2021b), implying that significant markers are likely in close physical proximity to causal variants. The Boosted Tree model for 8-leaf stage resistance highlighted SNP S6_21593433, located 162,184 bp from *Sobic.006G040401*, which encodes a zinc finger CCHC-type protein, and SNP S2_5624818, which was also identified by the Bootstrap Forest model (Fig. 5D; Table 2). The recurrence of S2_5624818 across both 8-leaf stage models suggests its potential importance in later-stage resistance.

### Machine learning analysis of combined phenotype identifies overlapping and novel candidate genes

We performed a machine learning analysis using a combined phenotype to identify potential candidate genes associated with anthracnose resistance across both seedling and 8-leaf stages. This combined phenotype was calculated by multiplying the seedling and 8-leaf stage anthracnose resistance scores for each accession, aiming to capture consistent resistance across developmental stages. We again used Bootstrap Forest and Boosted Tree models, trained on 100% of the data, focusing on the explanatory power of the models due to the limitations discussed previously.

**Fig. 6:**
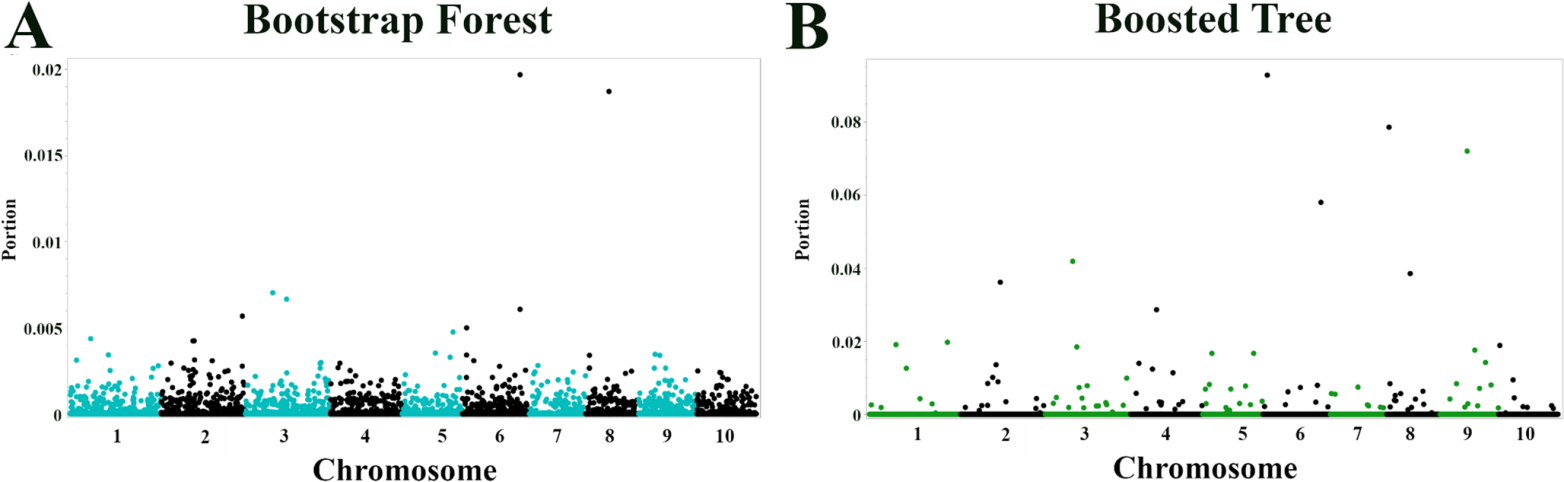
Genome-wide identification of SNPs associated with combined seedling and 8-Leaf stage anthracnose resistance using machine learning. (**A**) Bootstrap Forest and (**B**) Boosted Tree models for a combined anthracnose resistance phenotype (seedling score x 8-leaf score) in Senegalese sorghum. x-axis: Chromosomal position. y-axis: Importance score (proportion of contribution to model fit)

The Bootstrap Forest, applied to the combined phenotype, identified two SNPs with notable importance scores (Fig. 6; Table 3): S6_57126382, located within *Sobic.006G225800* (encoding an F-box domain protein), and S8_35974439, located 322,260 bp from *Sobic.008G090900* (encoding a protein with a DUF3411 domain).

**Table 3:** Genome-wide association of SNPs with combined seedling and 8-Leaf stage anthracnose resistance in sorghum, using bootstrap forest and boosted tree models. Manhattan plots show the snps’ genomic distribution associated with a combined anthracnose resistance phenotype (seedling score multiplied by 8-leaf score) in Senegalese sorghum. Results are shown for (a) bootstrap forest and (b) boosted tree models. x-axis: chromosomal position of each SNP. y-axis: importance score, reflecting the snp’s contribution to the model’s fit to the data. Distance definition: ‘0’ indicates the SNP is located within the gene (intragenic). Values > 0 indicate the distance in base pairs to the nearest annotated gene

SNP ID	Nearest/nearby gene and function	Base pairs away	Importance score (portion)
Bootstrap Forest			
S6_57126382	*Sobic.006G225800* F-box domain	0	0.02
S8_35974439	*Sobic.008G090900* DUF3411	322,260	0.19
Boosted Tree			
S6_2751834	*Sobic.006G017500* Leucine Rich Repeat	0	0.093
S8_1195771	*Sobic.008G014300* Mitochondrial glycoprotein	1,439	0.079
S9_36329620	*Sobic.009G098300* Uncharacterized protein *Sobic.009G098200* Putative PrMC3	18,845 20,149	0.072
S6_57126382	*Sobic.006G225800* F-box domain	0	0.058

The Boosted Tree model identified four SNPs with high importance scores (Fig. 6; Table 3): S6_2751834 (located within *Sobic.006G017500*, encoding a leucine-rich repeat protein), S8_1195771 (located within *Sobic.008G014300*, encoding a mitochondrial glycoprotein), S9_36329620 (located between *Sobic.009G098300*, an uncharacterized protein, and *Sobic.009G098200*, a putative PrMC3 protein), and S6_57126382. Notably, SNP S6_57126382 (associated with *Sobic.006G225800*, F-box domain protein) was identified by both models, suggesting a robust association with the combined resistance phenotype.

Furthermore, it’s essential to highlight the recurring importance of F-box domain-containing proteins. *Sobic.006G225800* was identified in both the Bootstrap Forest and Boosted Tree analyses of the combined phenotype and the Boosted Tree analysis of the seedling stage phenotype. The single-SNP association analysis also identified SNPs near *Sobic.001G272700* (F-box-like protein) as significantly associated with seedling stage resistance. Similarly, SNPs near genes encoding LRR domain-containing proteins (*Sobic.001G272800*, *Sobic.001G185000*, and *Sobic.006G017500*) were consistently identified across different analyses.

### Correlation analysis reveals limited overlap between machine learning and single-SNP approaches

**Fig. 7:**
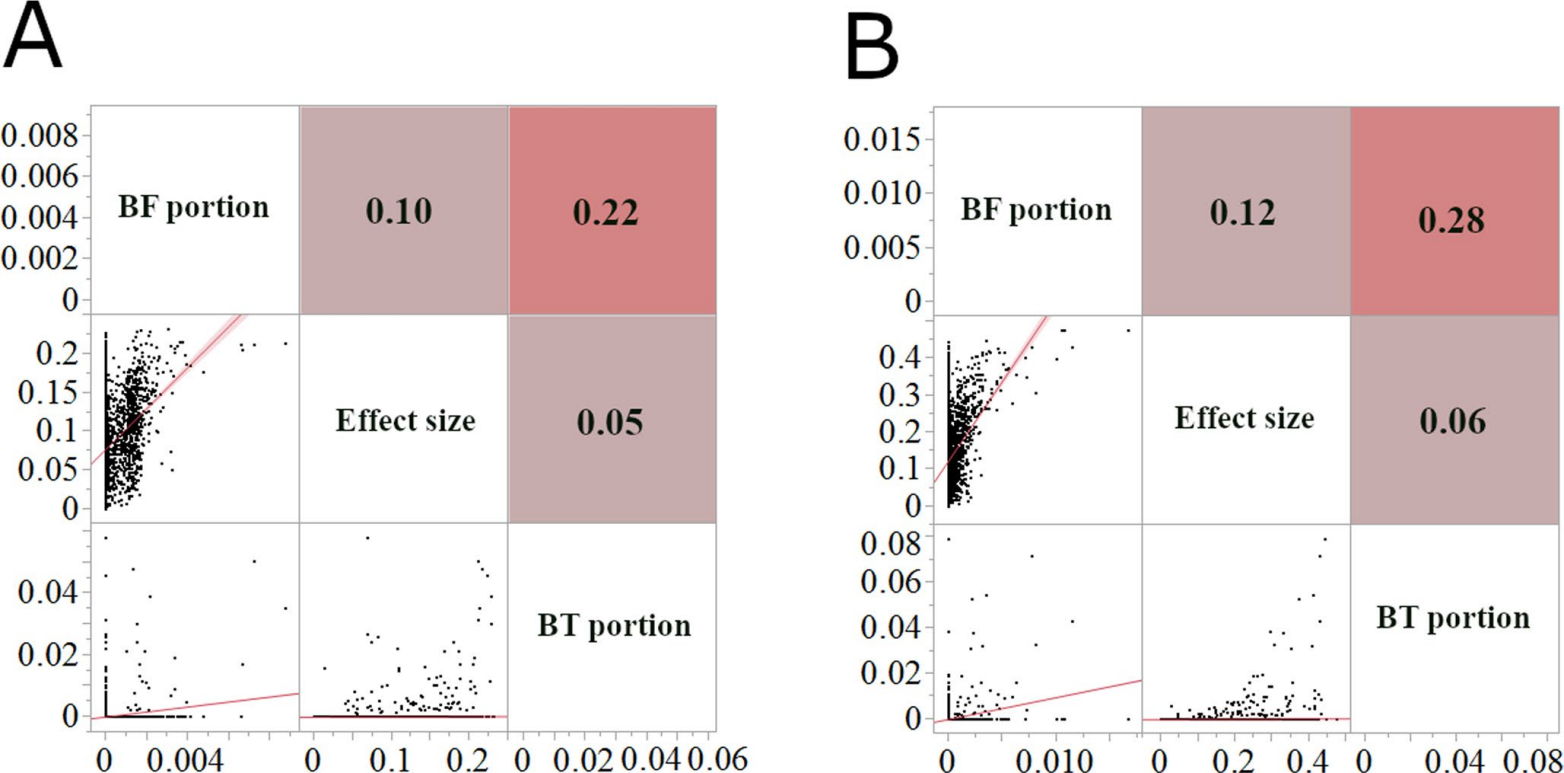
Correlation analysis of SNP importance/effect sizes across analytical methods for anthracnose resistance in sorghum. Pearson correlation coefficients (*r*) comparing SNP importance scores and effect sizes across different analytical methods. (**A**) Correlations between Bootstrap Forest (BF) importance scores, Boosted Tree (BT) importance scores, and single-SNP association analysis effect sizes for seedling stage anthracnose resistance. (**B**) Correlations between BF importance scores, BT importance scores, and single-SNP association analysis effect sizes for 8-leaf stage anthracnose resistance. All possible paired correlations were statistically significant (*p* < 0.0001) for both (a) & (b)

To assess the degree of concordance between the SNP rankings produced by the different analytical approaches, we performed a correlation analysis comparing the importance scores from the Bootstrap Forest and Boosted Tree models with the effect sizes from the single-SNP association analysis (Fig. 7). We performed this analysis separately for the seedling and 8-leaf stage data.

At the seedling stage, a moderate positive correlation (*r* = 0.47, R-squared = 0.22, *p* < 0.0001) was observed between the importance scores of the Bootstrap Forest and Boosted Tree models (Fig. 7 A), indicating some agreement in the SNPs identified as most important. However, correlations between the single-SNP association analysis effect sizes and the machine learning importance scores were weak (*r* = 0.12 with Bootstrap Forest effect size, and *r* = 0.07 with Boosted Tree, both *p* < 0.0001), although statistically significant. This indicates that the single-SNP and machine-learning methods are identifying largely distinct sets of SNPs. Methodologically, this weak correlation across the whole genome (*n* = 99,525) serves as quantitative evidence that the ensemble ML models are capturing non-linear genetic architectures and interaction effects that are mathematically distinct from the additive effects measured by the linear single-SNP analysis.

For the 8-leaf stage, a similar pattern was observed (Fig. 7B). The Bootstrap Forest and Boosted Tree importance scores showed a moderate positive correlation (*r* = 0.53, R-squared = 0.28, *p* < 0.0001). Correlations between the single-SNP association effect sizes and the machine learning importance scores were again weak, but statistically significant (*r* = 0.35 with Bootstrap Forest, and *r* = 0.24 with Boosted Tree; both *p* < 0.0001).

These results suggest that while there is some overlap in the SNPs identified as important by the different methods, the machine learning models, particularly Boosted Tree, capture information not captured by the single-SNP analysis, and vice versa. 

## Discussion

Sorghum anthracnose, caused by *C. sublineola*, substantially threatens sorghum production worldwide. While chemical control offers temporary relief, the rapidly evolving genetic resistance in fungal populations means that plant host resistance genes are the most sustainable and effective long-term solution. The previous studies identified SNPs potentially linked to the resistance to anthracnose at different stages of growth. When at the seedling stage, based on detached leaf assay, nearly all Senegalese genotypes were resistant, while at the 8-leaf stage, over 70 accessions (nearly half) scored near 3.0 on average, which leads to mild infection (Ahn et al. 2021b, 2022). Stage-dependent variations in infection response were also reported in johnsongrass (*Sorghum halepense*), which was significantly more resistant to anthracnose when at an early stage of life than later, potentially due to the cyanogenic glucoside dhurrin in sorghum species that is reported for its role in defense (Ahn et al. 2020; Fall et al. 2025).

In this study, we leveraged ML-based approaches, including Bootstrap Forest and Boosted Tree models, to identify novel SNPs associated with anthracnose resistance in Senegalese sorghum accessions, utilizing publicly accessible phenotypic (seedling and 8-leaf growth stages) data that would otherwise not be detectable with linear model-based association. Crucially, while preliminary analysis indicated limited predictive performance on validation sets, our findings are intended to be interpreted as purely explanatory. This approach emphasizes the Variable Importance Scores of SNPs to the model’s fit (e.g., Bootstrap Forest R^2^ of 0.78) to uncover biological candidates, rather than assessing the model’s capacity for phenotypic prediction on independent populations. While primarily explanatory due to limitations in independent validation, our analysis revealed several promising candidate genes, some of which were also supported by traditional single-SNP association analysis, suggesting their potential involvement in the complex genetic architecture of anthracnose resistance.

The most compelling evidence emerged for genes located on chromosome 1. Specifically, SNPs S1_52723450, S1_52723462, and S1_52723482, identified by both single-SNP analysis (Figs. 2 and 3; Table 1) and the Bootstrap Forest model in the seedling stage (Fig. 5; Table 2), are located in between *Sobic.001G272800*, encoding an LRR/disease resistance protein (RGA4), and *Sobic.001G272700*, encoding an F-box-like protein. Most disease resistance genes encode nucleotide-binding site leucine-rich repeat (NBS-LRR) proteins, which are involved in disease resistance in plants by recognizing effectors secreted by pathogens, directly or indirectly (McHale et al. 2006; Dubey and Singh 2018). In plant defense, F-box proteins constitute one of the largest families of proteins in plants, playing a crucial role in regulating various key physiological processes, including plant growth, development, and responses to external stimuli by specific recognition and ubiquitination of targets for degradation (Stefanowicz et al. 2015; Dubey and Singh 2018). No other annotated genes are present within nearby regions, suggesting that these SNPs, or variants in linkage disequilibrium with them, likely influence the expression or function of one or both of these genes, potentially leading to a synergistic effect in conferring resistance at the seedling stage. The Boosted Tree model identified *Sobic.003G430000* (aspartic peptidase) as highly influential in seedling resistance. Aspartic proteases are involved in various plant development functions. They are increasingly recognized for their role in plant-pathogen interactions, suggesting that localized cell death may play a role in limiting *C. sublineola* infection at the seedling stage (Figueiredo et al. 2021). The plant mediator complex (*Sobic.007G163100*), identified by the Boosted Tree model at the seedling stage, is central in coordinating plant adaptive responses and is critical for transcription regulation in plant defense and flowering pathways (An and Mou 2013; Yang et al. 2016; Zhai and Li 2019). Mannosylglycoprotein endo-beta-mannosidase (*Sobic.009G126000*) is a prominent candidate gene linked to resistance against multiple pathogens in a GWAS (Ahn et al. 2023). This gene has also been emphasized in seedling stage analyses using a Boosted Tree model and is recognized for its role in sugar metabolism and cell wall function (Mo and Bewley 2003; Dong et al. 2014; Garcia-Seco et al. 2017).

The biological relevance of these candidate genes identified by the ML models is further supported by transcriptomic evidence from previous studies on the sorghum-*C. sublineola* interaction. For instance, Natarajan et al. utilized RNA-Seq to profile gene expression in resistant and susceptible sorghum cultivars and identified significant differential expression in genes encoding LRR proteins and F-box domain-containing proteins upon infection (Natarajan et al. 2021). This aligns directly with our ML results, where SNPs near *Sobic.001G272800* (LRR) and *Sobic.001G272700* (F-box) were flagged as high-importance features. Furthermore, the detection of aspartic peptidase (*Sobic.003G430000*) by our Boosted Tree model is consistent with the known role of aspartyl proteases in mediating programmed cell death during plant defense responses, a mechanism also highlighted in the transcriptome analysis of sorghum inoculated with *C. sublineola* (Ahn et al. 2019; Natarajan et al. 2021).

At the 8-leaf stage, both the Bootstrap Forest and Boosted Tree models identified genes previously implicated in plant defense. Intriguingly, the LRR gene (*Sobic.006G274866*) was the only gene that overlapped between this ML-based analysis and traditional GWAS as a top candidate for anthracnose resistance. Additionally, other significant SNPs from both models were located in proximity to two genes on chromosome 2: *Sobic.002G058200*, encoding a Jasmonate O-methyltransferase, and *Sobic.002G058100*, encoding a Thioredoxin domain-containing protein, with the SNPs associated with the latter residing within its coding region.

An additional analysis of anthracnose resistance was performed using a phenotype representing the interaction between seedling and 8-leaf stage responses, calculated as the product of the two responses at different growth stages (Fig. 6; Table 3). The Bootstrap Forest model identified *Sobic.006G225800*, encoding an F-box domain-containing protein, and a SNP (S8_35974439) associated with *Sobic.008G090900*, encoding a protein with a DUF3411 domain with an unknown function, suggesting potential long-range regulation given the 322 kb distance. The Boosted Tree model identified *Sobic.006G017500*, which encodes an LRR protein; *Sobic.008G014300*, which encodes a mitochondrial glycoprotein homologous to a protein known to contribute to insecticide resistance in the cotton bollworm; and *Sobic.006G225800*, which encodes a protein containing an F-box domain.

To evaluate the ML approaches, we compared the SNP importance scores obtained from all three analyses (A single SNP association, Bootstrap Forest, and Boosted Tree) for both seedling and 8-leaf stage anthracnose resistance (Fig. 7). We observed statistically significant, but weak positive correlations at both stages across all possible pairs between the three models, implying that Bootstrap Forest and Boosted Tree capture distinct aspects of the genetic architecture. The Bootstrap Forest may focus more on major effect loci, while the Boosted Tree could uncover more subtle interactions. This interpretation is consistent with the fundamental mathematical properties of these ensemble methods: Bagging (Bootstrap Forest) primarily reduces variance, favoring robust signals, while Boosting (Boosted Tree) focuses on reducing bias, thereby effectively capturing more complex or subtle dependencies in the data (Elith et al. 2008; Hastie 2009).

A limitation of this study lies in the differing phenotyping methods employed for the seedling and 8-leaf stages. Seedling assays utilized detached leaves inoculated with a single, highly virulent *C. sublineola* isolate (FSP53). In contrast, 8-leaf stage assays used whole-plant inoculations in a greenhouse with a mixture of eight isolates, including FSP53, among the most virulent strains (Ahn et al. 2021b). Although this difference in methodology introduces a potential confounding factor, research by Ahn et al. suggests that when sorghum accessions are challenged with mixtures of *C. sublineola* isolates varying in virulence, the disease response tends to be driven by the most virulent isolate present (Ahn et al. 2021a). This supports the idea that including the highly virulent FSP53 isolate in the 8-leaf stage mixture likely played a dominant role in determining the overall disease response. Therefore, while we acknowledge the methodological differences, the genes identified in our analysis, particularly those highlighted by the combined-stage phenotype (Table 3), may still represent essential factors in anthracnose resistance across the sorghum life cycle. However, further research, ideally using consistent phenotyping methods across developmental stages, is necessary to definitively confirm the role of these genes in conferring durable, broad-spectrum resistance.

Despite the limitations, our study demonstrates the potential of ML to accelerate the discovery of candidate resistance genes in sorghum. The identified genes, particularly with known or plausible roles in plant defense, represent promising targets for future functional validation. The next crucial steps involve confirming the role of these genes through targeted experiments, such as gene editing or overexpression studies, and evaluating their contribution to resistance in diverse genetic backgrounds and under different environmental conditions. Additionally, developing molecular markers associated with the identified SNPs could enhance marker-assisted selection (MAS) in sorghum breeding programs, improving the efficiency and precision of traditional plant breeding and speeding up the introgression of anthracnose resistance into elite cultivars (Ribaut and Hoisington 1998; Collard and Mackill 2008). This, in turn, could contribute to more sustainable sorghum production, reducing reliance on chemical control and enhancing food security, particularly in regions where sorghum is a staple crop.

## Conclusion

This study utilized Bootstrap Forest and Boosted Tree models, making it possible to understand a new genetic architecture of anthracnose resistance in Senegalese sorghum, considering both seedling and 8-leaf stages and a combined resistance phenotype. While limitations in model validation necessitate a cautious interpretation, our findings highlight several promising genomic regions and genes. The recurring identification of genes related to LRR-mediated defense and F-box protein function across different analytical approaches and developmental stages suggests their potentially crucial role in sorghum’s response to *C. sublineola*. Overall, this study offers valuable information for future research in plant breeding to enhance anthracnose resistance in sorghum and a broad range of crops.

## Data Availability

We used publicly available data for both phenotypic and genotypic data. Additional details regarding machine learning results can be provided upon request to the corresponding authors.
